# Circulating miR-99a-5p and miR-1246 as Diagnostic and Stage-Associated Biomarkers in Laryngeal Squamous Cell Carcinoma

**DOI:** 10.3390/biomedicines14030659

**Published:** 2026-03-13

**Authors:** Alexandru-Romulus Hut, Gheorghe Iovanescu, Eugen Radu Boia, Delia Ioana Horhat, Andrada Ioana Dumitru, Mihail Alexandru Badea, Catalin Marian, Paula Diana Ciordas, Nicolae Constantin Balica

**Affiliations:** 1 Doctoral School, “Victor Babes” University of Medicine and Pharmacy, Eftimie Murgu Square 2, 300041 Timisoara, Romania; alexandru.hut@umft.ro (A.-R.H.); andrada.dumitru@umft.ro (A.I.D.); balica@umft.ro (N.C.B.); 2 Department of Ear-Nose-Throat, Faculty of Medicine, “Victor Babes” University of Medicine and Pharmacy, Eftimie Murgu Square 2, 300041 Timisoara, Romania; giovanescu@umft.ro (G.I.); boia.eugen@umft.ro (E.R.B.); horhat.ioana@umft.ro (D.I.H.); 3 Dermatology Department, The George Emil Palade University of Medicine, Pharmacy, Science, and Technology, 540139 Targu Mureș, Romania; 4 Department IV, Biochemistry and Pharmacology, “Victor Babes” University of Medicine and Pharmacy, Eftimie Murgu Square 2, 300041 Timisoara, Romania; cmarian@umft.ro

**Keywords:** laryngeal neoplasms, microRNAs, biomarkers, liquid biopsy, polymerase chain reaction

## Abstract

**Background and Objectives**: Circulating microRNAs may provide minimally invasive biomarkers for laryngeal squamous cell carcinoma (LSCC), but clinically interpretable data for miR-99a-5p and miR-1246 remain limited. We compared circulating levels of these two miRNAs between LSCC patients and controls and explored stage-associated differences within the cancer cohort. **Methods**: This single-center case–control study was conducted in Timișoara, Romania. Circulating miRNAs were quantified by RT-qPCR. Expression was summarized as ΔCt [Ct(target) − Ct(miR-16)] and as the relative expression (2^−ΔΔCt^) using the control group as a calibrator. Group comparisons used Mann–Whitney U tests, associations used Spearman correlation, and the diagnostic performance was assessed by ROC analysis and multivariable logistic regression. **Results**: Fourteen controls were compared with cancer patients with available miRNA measurements (miR-99a-5p, *n* = 53; miR-1246, *n* = 49). miR-99a-5p showed significantly higher ΔCt values in cancer patients than in the controls (5.308 [IQR 4.139–6.864] vs. 3.184 [2.142–3.708], *p* < 0.001), corresponding to a lower relative expression (fold-change 0.200 [0.068–0.449], *p* < 0.001). miR-1246 did not differ significantly between cancer and controls (*p* = 0.09). Within the cancer cohort, advanced-stage disease showed a lower relative miR-1246 expression than early-stage disease (ΔCt 5.820 [4.502–6.972] vs. 4.233 [3.109–5.372], *p* = 0.01; fold-change 0.363 vs. 1.091, *p* = 0.01), while miR-99a-5p showed a non-significant difference in the same direction (*p* = 0.052). miR-99a-5p discriminated cancer patients from the controls with an AUC of 0.842 (95% CI 0.744–0.931), sensitivity of 77.4%, and specificity of 92.9% at ΔCt = 4.018. In multivariable analysis, ΔCt(miR-99a-5p) remained independently associated with cancer status (OR 1.89, 95% CI 1.19–3.00; *p* = 0.007). **Conclusions**: Circulating miR-99a-5p showed the strongest diagnostic signal in LSCC, whereas miR-1246 appeared more informative for stage-associated biological stratification.

## 1. Introduction

Laryngeal cancer remains a clinically consequential head-and-neck malignancy, because it directly compromises airway protection, voice, swallowing, and social functioning. In 2020, the global cancer burden was estimated at 19.3 million new cases and ~10.0 million deaths, underscoring the scale at which site-specific cancers contribute to morbidity and mortality worldwide [1]. Contemporary population-level analyses further suggest that there is a substantial geographic variability in laryngeal cancer burden and the continued influence of modifiable exposures (notably tobacco and alcohol), alongside broader metabolic and environmental risk profiles [2]. Despite refinements in organ-preservation and multimodality care, prognoses still diverge markedly by stage, with large clinical cohorts showing steep survival gradients from early to advanced disease [3]. These observations emphasize that, while TNM staging remains essential for treatment planning, it cannot fully account for the underlying biological heterogeneity that drives clinical variability across anatomically similar tumors [4].

In this context, there is increasing interest in biomarkers that better capture tumor behavior beyond anatomy and provide actionable stratification across clinically relevant subgroups (nodal disease, early vs. advanced stage, and treatment exposure). MicroRNAs (miRNAs) are particularly attractive candidates because they regulate post-transcriptional gene expression programs implicated in proliferation, apoptosis, invasion, immune modulation, and therapy response. In laryngeal cancer specifically, evidence syntheses describe a rapidly expanding but methodologically heterogeneous literature across tissue, serum/plasma, and extracellular vesicle (EV) compartments, suggesting a meaningful biological signal yet highlighting the need for careful validation and clinically anchored analyses [5]. More recent systematic work focused on circulating miRNAs with independent validation similarly indicates that reproducible candidates may exist, but translation requires standardized specimen handling, normalization, and reporting to reduce between-study variability [6].

Blood-based (“liquid”) biomarkers are particularly valuable in laryngeal oncology, because repeated tumor sampling is often impractical, and longitudinal monitoring could support the earlier detection of recurrence, response assessment, and treatment tailoring. EVs (including exosomes) provide a biologically plausible mechanism for biomarker enrichment because they can transport small RNAs between cells and potentially reflect tumor–microenvironment crosstalk [7]. Independently, circulating miRNAs have been demonstrated to be detectably stable in blood-based matrices, enabling minimally invasive measurements and supporting biomarker development efforts [8]. However, pre-analytical and analytical variability remains a major pitfall: blood cell-derived miRNAs and hemolysis can substantially confound circulating measurements, reinforcing the need for rigorous processing, quality control, and interpretation, especially in case–control designs and subgroup analyses [9].

Among candidate markers, miR-99a-5p has frequently been characterized as tumor-suppressive across solid tumors, often converging on growth and stress-response signaling nodes, making reduced expression a biologically plausible indicator of malignant transformation or aggressive behavior. In head-and-neck squamous cell carcinoma, EV-derived miRNA profiling has identified plasma EV miR-99a-5p among candidates with diagnostic promise, supporting the continued evaluation of this miRNA in clinically defined cohorts [10]. Complementing these findings, tissue-based profiling work in laryngeal cancer has reported miRNA signatures associated with nodal metastasis, reinforcing the concept that miRNA dysregulation may coincide with clinically meaningful progression phenotypes and motivating analyses stratified by nodal status and stage [11].

miR-1246 has also attracted attention in cancer biomarker research because it is frequently linked to tumor progression and inflammation-related signaling, yet its directionality may depend on the disease context and biological compartment (tumor vs. circulation; vesicle-associated vs. non-vesicular). In laryngeal squamous cell carcinoma, exosome-focused mechanistic work implicates the exosomal miR-1246 in tumor–immune interactions, including pathways related to macrophage polarization that can plausibly facilitate invasion and dissemination [12]. Adding complexity, exosomal miR-1246 biology may involve non-canonical processing (including reports of derivation from RNU2-1), which has potential implications for assay interpretation across platforms and compartments [13]. Consistent with a pro-metastatic role in head-and-neck contexts, exosomal miR-1246 has also been shown to promote motility and invasion in oral squamous cell carcinoma models via regulation of invasion-related targets, further supporting its candidacy as a progression-associated signal [14].

From a methodological perspective, RT-qPCR remains a pragmatic platform for circulating miRNA quantification because it is widely available, analytically sensitive, and compatible with standardized reporting. Relative quantification using the comparative Ct method (ΔΔCt) provides a transparent framework for reporting fold-changes, anchored to an endogenous reference (here, miR-16) and a calibrator group (controls) [15]. Building on these biological and practical considerations, the present study was designed to (i) compare circulating miR-99a-5p and miR-1246 between laryngeal cancer patients and controls, (ii) evaluate whether expression differs by stage (early I–II vs. advanced III–IV), (iii) explore clinically relevant subgroups (nodal disease and systemic therapy exposure), and (iv) quantify associations with age and comorbidity burden, including diagnostic performance estimates.

Although both miR-99a-5p and miR-1246 have previously been implicated in head-and-neck tumor biology, their circulating behavior in clinically stratified laryngeal squamous cell carcinoma remains insufficiently defined. Prior studies have often mixed anatomic subsites, focusing on extracellular vesicle-enriched fractions rather than whole circulating fractions or emphasizing discovery signatures rather than clinically interpretable RT-qPCR analyses within staged LSCC cohorts. As a result, it remains unclear whether these two biologically distinct miRNAs provide complementary value for diagnosis versus within-cancer stratification. The present study was therefore designed to evaluate circulating miR-99a-5p and miR-1246 in histologically confirmed LSCC and non-cancer controls using a uniform RT-qPCR approach, with pre-specified analyses across stages, nodal disease, treatment exposure, and multimarker discrimination.

## 2. Materials and Methods

### 2.1. Study Design, Setting, and Participants

This observational single-center case–control study was conducted within the university-affiliated clinical network of Victor Babeș University of Medicine and Pharmacy, in collaboration with the Otorhinolaryngology/Head-and-Neck Oncology services of Pius Brînzeu Clinical Emergency Hospital, Timișoara, Romania, between March 2022 and October 2024. The primary objective was to compare circulating miR-99a-5p and miR-1246 expressions between patients with laryngeal squamous cell carcinoma (LSCC) and non-cancer controls. Secondary objectives were to evaluate differences according to disease stage, nodal status, and treatment exposure.

Eligible cases were adults aged ≥18 years with histopathologically confirmed LSCC and an available blood sample of adequate quality for RT-qPCR analysis. Tumor stage was recorded according to the AJCC/UICC TNM classification, as documented in the medical records by the treating team, and was grouped as early stage (I–II) or advanced stage (III–IV). Exclusion criteria for cancer cases were non-squamous histology; prior head-and-neck malignancy or synchronous second primary cancer; active infection or acute inflammatory condition at the time of blood sampling; autoimmune flare or hematologic disorder likely to affect circulating RNA measurements; recent transfusion within 4 weeks; inadequate biospecimen volume or quality; and missing essential clinicopathologic or molecular data. Controls were adults without current or previous malignancy, without active infection or acute inflammatory disease at sampling, and without suspicious upper aerodigestive tract lesions on routine ENT clinical evaluation.

The term “consecutive patients” means that all eligible LSCC patients presenting during the predefined recruitment interval were screened in chronological order and invited to participate without additional selection based on tumor stage, nodal status, treatment status, or molecular result. Sex was recorded for all participants.

Blood was collected at study enrollment. In newly diagnosed cases, sampling was performed before initiation of a new oncologic treatment line. In previously treated patients undergoing routine reassessment, blood was obtained at scheduled follow-up visits, at least 4 weeks after the last chemotherapy administration, and 8 weeks after completion of radiotherapy to reduce the influence of immediate post-treatment fluctuations on circulating miRNA measurements.

### 2.2. Biospecimen Workflow and RT-qPCR Measurement Framework

miR-16 was selected a priori as the endogenous reference because it was consistently amplified across the cohort and showed low dispersion in an internal stability check performed on a pilot subset of samples (10 controls and 10 cases), with no significant case–control difference in Ct values (median 22.7 [IQR 22.2–23.1] vs. 22.8 [22.3–23.4], *p* = 0.64). Nevertheless, because circulating miR-16 may still be influenced by blood cell contamination, this normalization choice is acknowledged as a study limitation.

Peripheral venous blood (6 mL) was collected into K2EDTA Vacutainer tubes (Becton Dickinson, Franklin Lakes, NJ, USA; Cat. No. 367863) and processed within 2 h of venipuncture. Samples were centrifuged at 1900× *g* for 10 min at 4 °C to obtain plasma, followed by a second centrifugation at 16,000× *g* for 10 min at 4 °C to reduce residual cellular contamination. Cell-free plasma was aliquoted into RNase-free tubes (500 μL aliquots) and stored at −80 °C until extraction. Samples subjected to more than one freeze–thaw cycle were not analyzed.

Visible hemolysis was assessed after the second centrifugation, and a spectrophotometric screening step was performed at 414 nm. Samples with visible pink/red discoloration or A414 > 0.20 were excluded from analysis. Although this does not replace a dedicated hemolysis-adjusted normalization strategy, it reduced the likelihood of marked free-hemoglobin contamination.

Total RNA enriched for small RNAs was extracted from 200 μL plasma using the miRNeasy Serum/Plasma Advanced Kit (QIAGEN, Hilden, Germany; Cat. No. 217204) according to the manufacturer’s instructions and eluted in 20 μL RNase-free water. An exogenous spike-in control was not used in the present analysis; the previous wording only referred to a general methodological option and has now been removed to avoid ambiguity.

Reverse transcription was performed using the TaqMan Advanced miRNA cDNA Synthesis Kit (Applied Biosystems, Thermo Fisher Scientific, Waltham, MA, USA; Cat. No. A28007). The workflow included poly(A) tailing at 37 °C for 45 min followed by 65 °C for 10 min, adaptor ligation at 16 °C for 60 min, reverse transcription at 42 °C for 15 min followed by 85 °C for 5 min, and an miR-Amp pre-amplification step at 95 °C for 5 min, then 14 cycles of 95 °C for 3 s and 60 °C for 30 s, followed by 99 °C for 10 min. The pre-amplified product was diluted 1:10 in 0.1× TE buffer before qPCR.

Quantitative PCR was run on a QuantStudio 5 Real-Time PCR System (Applied Biosystems) using TaqMan Fast Advanced Master Mix. Each reaction had a final volume of 20 μL and contained 10 μL 2× master mix, 1 μL 20× TaqMan Advanced miRNA Assay, 4 μL nuclease-free water, and 5 μL diluted cDNA template. Thermal cycling was performed in fast mode as follows: 95 °C for 20 s, then 40 cycles of 95 °C for 1 s and 60 °C for 20 s. All reactions were run in triplicate, and no-template controls were included in each assay plate. Replicate sets with a within-sample SD > 0.35 Ct were repeated, and assays with Ct > 35 in at least two replicates were considered below reliable detection and treated as missing.

The analyzed targets were hsa-miR-99a-5p, hsa-miR-1246, and hsa-miR-16-5p as endogenous references. Because the commercial hydrolysis probe assays use proprietary primer/probe chemistry, primer sequences are not publicly disclosed by the manufacturer; therefore, mature miRNA identifiers, accessions, and mature sequences are now reported in Table 1.

### 2.3. Variables, Endpoints, and Study Groups

Clinical variables were as follows: age (years), residence (urban vs. rural), TNM elements (T, N, and M) parsed from the recorded TNM string, and derived stage group defined as early (I–II) vs. advanced (III–IV). Nodal disease was defined as N+ (N1–N3) vs. N0, and T4 status was recorded as T4 vs. non-T4. Treatment indicators were extracted as “yes/no” fields for radiotherapy and chemotherapy; missing entries were retained as missing rather than imputed. Comorbidity burden was summarized as a comorbidity count across the available binary comorbidity flags (hypertension and other recorded conditions).

Molecular endpoints: For each miRNA target, ΔCt was computed as Ct(target) − Ct(miR-16). Relative expression vs. controls used the comparative method, ΔΔCt = ΔCt(sample) − meanΔCt(controls), with fold-change = 2^−ΔΔCt^. This yields an interpretable estimate where values < 1 indicate downregulation vs. controls and values > 1 indicate upregulation.

The primary comparisons were as follows: (1) controls vs. cancer (case–control); (2) early vs. advanced stage among cancer patients. Secondary subgroup comparisons included chemotherapy-exposed vs. not exposed (within cancer) and N+ vs. N0 (within cancer). Correlation analyses assessed associations between miRNA ΔCt values and age, stage number, and comorbidity count.

### 2.4. Statistical Analysis

Statistical comparisons were based primarily on ΔCt values because these preserve the qPCR measurement scale and are generally more suitable for inference than ratio-transformed relative expression values. For biological interpretability, 2^−ΔΔCt^ fold-change values relative to the control calibrator were also reported. Higher ΔCt values indicate lower relative abundance of the miRNA target.

All tests were two-sided with α = 0.05. Continuous variables were summarized as mean ± SD when approximately symmetric (e.g., age) and as median [IQR] for Ct-derived metrics and fold-changes (typically skewed). Categorical variables were summarized as *n*/N (%), with N reflecting non-missing observations for that field.

Between-group comparisons for categorical variables used χ^2^ tests or Fisher’s exact tests when expected cell counts were small. For Ct-derived metrics (ΔCt and fold-change), we used Mann–Whitney U tests because distributional assumptions for parametric testing are often violated in qPCR-derived endpoints. Associations were evaluated with Spearman rank correlations (ρ). Diagnostic discrimination (control vs. cancer) was quantified using ROC AUC with bootstrap 95% confidence intervals and the Youden index to identify an optimal ΔCt threshold (reported descriptively).

## 3. Results

Baseline clinical and demographic characteristics stratified by stage are summarized in Table 2 and Table 3. Compared with early-stage disease, advanced-stage LSCC was associated with substantially higher frequencies of T4 tumors and node-positive disease. By contrast, age, sex, residence, radiotherapy exposure, chemotherapy exposure, and comorbidity burden did not differ significantly between stage groups.

In the case–control analysis, the circulating miR-99a-5p was significantly lower in cancer patients than in the controls, whereas miR-1246 did not differ significantly between groups (Table 4).

Within the cancer cohort, advanced-stage disease was associated with lower relative circulating miR-1246 expression than early-stage disease. miR-99a-5p showed a non-significant difference in the same direction (Table 5).

Patients who had received chemotherapy showed lower miR-99a-5p fold-change values than those without chemotherapy exposure, whereas no significant difference was observed for miR-1246.

The two circulating miRNAs were moderately correlated with each other in cancer patients, but neither marker correlated significantly with age, stage, or comorbidity burden.

ROC analysis demonstrated a strong discriminative ability for miR-99a-5p ΔCt (controls *n* = 14; cancer *n* = 53), yielding an AUC of 0.842 with bootstrap 95% CI [0.744, 0.931]. Using the Youden-optimal cutoff ΔCt = 4.018, the sensitivity was 77.40% and specificity was 92.90%, consistent with robust case–control separation (*p* < 0.001). In comparison, miR-1246 ΔCt (controls *n* = 14; cancer *n* = 49) showed a weaker performance (AUC 0.65; 95% CI [0.498, 0.782]), with an optimal cutoff of ΔCt = 5.344, providing a 59.20% sensitivity and 71.40% specificity, aligning with the non-significant group difference (*p* = 0.09), as seen in Table 6.

In the multivariable logistic regression model (N = 59; 45 cancer and 14 controls), ΔCt(miR-99a) was a statistically significant predictor of cancer status (β = 0.635, SE = 0.236), corresponding to an OR of 1.89 (95% CI 1.19–3.00; *p* = 0.007), indicating higher odds of cancer with increasing ΔCt (lower expression). ΔCt(miR-1246) was not independently associated with case status (β = 0.111, SE = 0.195; OR 1.12 [0.76–1.64]; and *p* = 0.57), as seen in Table 7.

The suppression score differed significantly between stage groups, with early-stage cancer (I–II; *n* = 12) showing a negative mean score (−0.675 ± 0.921) and advanced-stage cancer (III–IV; *n* = 31) showing a positive mean score (0.148 ± 1.126). The mean difference (advanced minus early) was 0.823 (95% CI 0.135–1.512), reaching statistical significance (*p* = 0.021, Welch’s *t*-test) and reflecting a moderate-to-large standardized effect size (Hedges g = 0.752), consistent with a meaningful shift in the composite suppression metric across stages (Table 8).

In robust regression Model A (N = 43), stage_num was positively associated with the suppression score (β = 0.643, SE = 0.294; *p* = 0.029), indicating higher scores with increasing stages, while T4 status showed an inverse association (β = −1.061, SE = 0.528; *p* = 0.044). Node-positive disease, age, and comorbidity_count were not significant predictors (all *p* > 0.40). In the exploratory interaction Model B (N = 19, chemotherapy recorded), neither the chemotherapy main effect (β = −8.579, SE = 10.377; *p* = 0.408) nor the stage_num × chemotherapy interaction (β = 2.250, SE = 3.248; *p* = 0.489) was significant, and none of the covariates reached significance (all *p* ≥ 0.145), suggesting limited power and/or weak interaction effects in the reduced subset (Table 9, Table 10 and Table 11).

**Figure 1:**
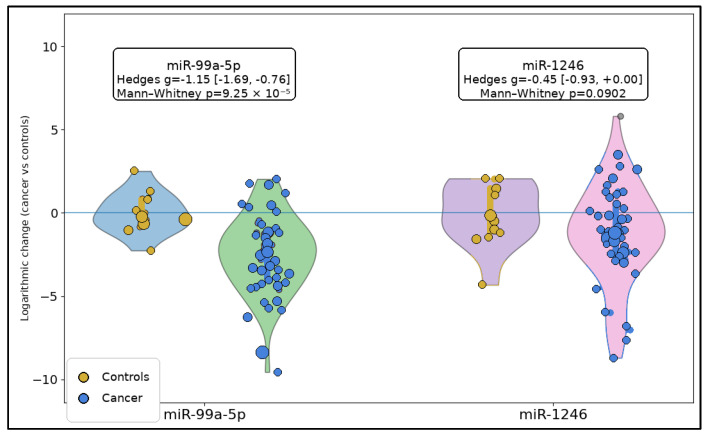
Raincloud plots of log2 fold-change distributions for miR-99a-5p and miR-1246 in controls and laryngeal squamous cell carcinoma. Each panel includes a half-violin density plot, an embedded boxplot showing the median and interquartile range, and jittered individual observations. Sample sizes were as follows: miR-99a-5p, controls *n* = 14 and cancer *n* = 53; miR-1246, controls *n* = 14 and cancer *n* = 49.

Across the qPCR-derived log2 fold-change (2^−ΔΔCt^) distributions, miR-99a-5p showed a clear and large separation between groups: cancer samples were shifted toward more negative log2FC values (greater downregulation vs. calibrator) compared with the controls, with a large standardized effect (Hedges g = −1.15; 95% CI −1.69 to −0.76) and a statistically significant difference (Mann–Whitney *p* = 9.25 × 10^−5^). In contrast, miR-1246 demonstrated substantially greater dispersion and overlap between groups, yielding only a modest effect that did not reach conventional significance (Hedges g = −0.45; 95% CI −0.93 to 0.00; and *p* = 0.0902), as seen in Figure 1.

**Figure 2:**
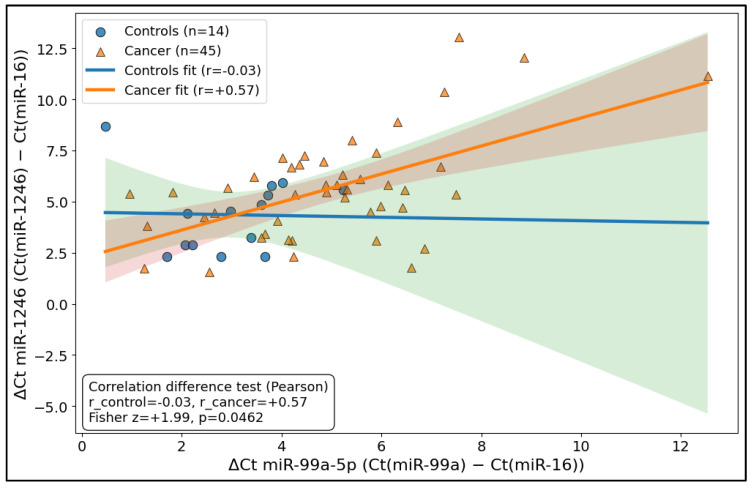
Differential coupling plot (control vs. cancer “miRNA–miRNA relationship”).

The relationship between the two miRNAs differed markedly by disease state: in controls (*n* = 14), ΔCt values for miR-99a-5p and miR-1246 were essentially uncorrelated (r = −0.03), whereas in cancer samples (*n* = 45), the same pair showed a moderate positive coupling (r = 0.57), consistent with coordinated behavior or shared variance emerging in the malignant context. Importantly, the difference in correlation strength between groups was statistically supported using Fisher’s *z* test (z = 1.99, *p* = 0.0462), indicating that cancer status is associated not only with the shifts in expression levels but also with a change in the miRNA–miRNA dependency structure (Figure 2).

**Figure 3:**
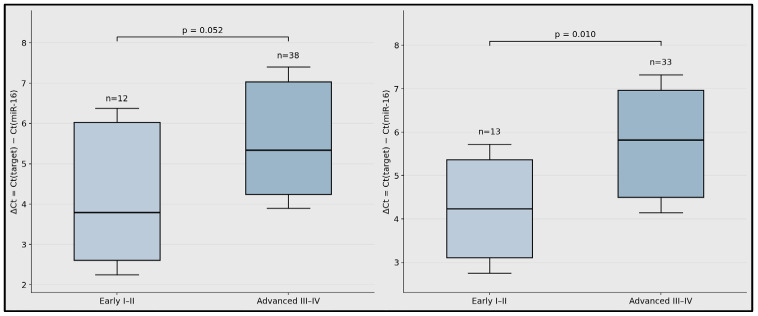
Stage-stratified circulating miRNA expression in laryngeal squamous cell carcinoma.

Summary box–interval plots show ΔCt distributions for miR-99a-5p and miR-1246 according to stage group. Higher ΔCt values indicate a lower relative circulating expression. Sample sizes were as follows: miR-99a-5p, early-stage *n* = 12 and advanced-stage *n* = 38; miR-1246, early-stage *n* = 13 and advanced-stage *n* = 33 (Figure 3).

**Figure 4:**
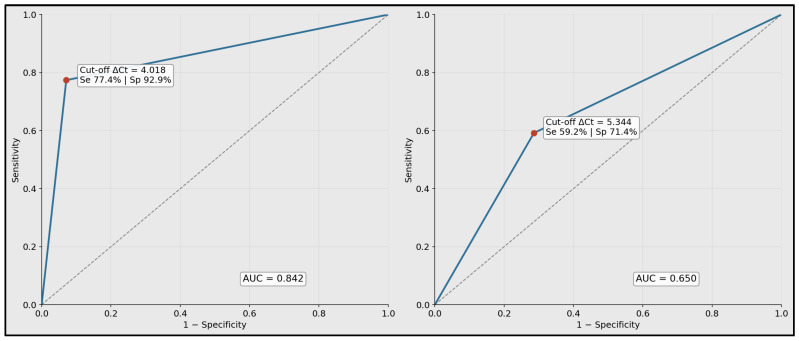
Receiver operating characteristic curves for discrimination of laryngeal squamous cell carcinoma versus controls.

ROC plots are shown for miR-99a-5p and miR-1246 using ΔCt values as predictors. The annotated operating points correspond to the Youden-optimal thresholds reported in Table 6. The plotted summary curves reproduce the reported AUC and threshold performance characteristics (Figure 4).

## 4. Discussion

### 4.1. Analysis of Findings

The present study demonstrates a marked downregulation of circulating miR-99a-5p in laryngeal cancer relative to controls, with a large separation in ΔCt and fold-change distributions and strong diagnostic performance (AUC 0.842, sensitivity 77.4%, and specificity 92.9%). This magnitude is directionally consistent with prior LSCC liquid biopsy work showing that circulating miRNAs can discriminate cancer from healthy comparators, although the specific candidate sets and normalization approaches differ substantially across reports [16,17,18]. From a biological standpoint, miR-99a-5p has been described as predominantly tumor-suppressive in head-and-neck squamous cell carcinoma (HNSCC), supporting the plausibility that reduced circulating levels may reflect malignant transformation and/or tumor burden [19]. In parallel, extracellular vesicle (EV) investigations in head-and-neck cancer have also identified EV-derived miR-99a-5p as diagnostically informative, suggesting that tumor-associated packaging and release may contribute to the detectability of this signal in blood-based matrices [20].

The control group was relatively small and not fully characterized for several major LSCC-related confounders. Although controls were selected to exclude known malignancy and acute inflammatory illness at the time of sampling, residual confounding by factors such as sex, tobacco exposure, alcohol use, and chronic inflammatory status remains possible. This limitation is particularly relevant in case–control biomarker research, where baseline clinical differences may influence circulating miRNA distributions independently of cancer status.

Within-case stratification suggested phenotype-dependent miRNA behavior. miR-1246 showed a significantly lower relative expression in advanced (III–IV) versus early (I–II) disease, while miR-99a-5p was non-significant. These findings are consistent with the broader EV-miRNA literature in HNSCC, which emphasizes that circulating signals may vary by tumor stage, tumor–microenvironment interactions, and the biological compartment measured (total plasma/serum vs. EV-enriched fractions), with meaningful differences in the apparent effect size across analytic workflows [20,21]. Mechanistically, interpretation of miR-1246 requires particular caution, because “exosomal miR-1246” signals can originate from RNU2-1 fragments via non-canonical processing, which can influence assay specificity and cross-platform concordance [22]. Additionally, miR-1246 has been linked to oncogenic phenotypes and treatment resistance (e.g., via CCNG2-related pathways), supporting its biological relevance even when bulk circulating measurements show a wide dispersion and partial overlap between groups [23].

Treatment subgroup analyses indicated that chemotherapy exposure was associated with a stronger suppression of miR-99a-5p, whereas miR-1246 did not differ significantly by chemotherapy status. Prior studies in head-and-neck cancer have shown that circulating miRNAs can be therapy-responsive, changing during radiotherapy and correlating with prognosis and treatment response, supporting the concept that some circulating miRNAs function as dynamic biomarkers rather than static diagnostic markers [24,25]. However, chemotherapy exposure is commonly intertwined with disease severity (advanced stage and higher-risk features), and the present exploratory interaction modeling was limited by a reduced sample size among patients with recorded chemotherapy. Therefore, the observed association is best interpreted as hypothesis-generating and may reflect a composite of tumor aggressiveness, systemic treatment exposure, and host response rather than a direct pharmacologic effect alone [24,25].

Beyond level differences, the study identified a cancer-associated change in the miRNA–miRNA dependency structure, with miR-99a-5p and miR-1246 shifting from no measurable association in controls to moderate coupling in cancer. Such “network-level” behavior is biologically plausible in malignancy, where coordinated transcriptional programs, shared upstream regulation, and EV-mediated co-release can generate emergent dependencies that are not present in non-cancer states [21]. This observation complements the notion that multi-feature representations (including correlation structure) may capture disease biology not fully reflected by univariate shifts, and it supports the further evaluation of combined biomarkers and model-based approaches in larger, independently validated cohorts [18,21].

Finally, multivariable modeling indicated that miR-99a-5p carried the dominant independent diagnostic contribution, whereas miR-1246 added limited incremental value for case–control discrimination in this dataset—consistent with its weaker standalone AUC and greater dispersion. Recent LSCC serum profiling has similarly suggested that panels can achieve high selectivity/specificity, but also illustrates that reported performance depends heavily on cohort structure, pre-analytics, and internal vs. external validation [18]. Collectively, these results support prioritizing miR-99a-5p as a streamlined candidate for diagnostic development, while positioning miR-1246 as a context-dependent marker that is potentially more informative for stage biology, EV-enriched assays, or longitudinal monitoring, provided that pre-analytical control and assay specificity are rigorously standardized [20,21,22,23].

If validated externally, circulating miR-99a-5p could be integrated as a minimally invasive adjunct to clinical evaluation to improve case–control discrimination (AUC 0.842; 77.4% sensitivity and 92.9% specificity at ΔCt = 4.018) and potentially support risk-refined triage when endoscopy/imaging pathways are constrained. miR-1246 demonstrated a stage-associated signal (advanced vs. early: ΔCt 5.820 vs. 4.233; *p* = 0.01), suggesting potential utility for biological stratification within confirmed cancer (e.g., complementing TNM to flag higher-burden disease biology). The observed, stronger chemotherapy-associated downregulation of miR-99a-5p (fold-change 0.135 vs. 0.438; *p* = 0.017) also motivates prospective studies evaluating these markers as treatment-monitoring or response-associated endpoints.

From a clinical perspective, the principal value of the present findings lies in the potential separation of two biomarker functions. Circulating miR-99a-5p showed the clearest case–control discrimination and may therefore be more relevant as a minimally invasive diagnostic adjunct, whereas miR-1246 appeared more informative for within-cancer biological stratification by stage. This distinction is clinically useful, because biomarkers do not necessarily serve the same purpose across the disease pathway. At the same time, these implications must be interpreted cautiously. The small control group limits the precision of reference distributions and ROC thresholds, while the small early-stage and chemotherapy-exposed subgroups reduce power for subgroup analyses and increase the risk of unstable effect estimates. Accordingly, the present study should be viewed as hypothesis-generating and supportive of larger validation cohorts rather than definitive for immediate clinical implementation.

### 4.2. Study Limitations

This analysis is limited by its single-center design and small control sample, which reduce the precision of reference distributions and ROC threshold estimates. Missingness in treatment variables, particularly chemotherapy exposure, reduced the sample size for subgroup and interaction analyses and may have introduced selection bias. The study was cross-sectional and did not include longitudinal sampling, recurrence endpoints, or survival outcomes. In addition, circulating miRNA measurements are sensitive to pre-analytical variability, possible hemolysis or blood cell contamination, and the choice of endogenous normalizer. In the present cohort, miR-16 was used as the reference miRNA, but formal validation against multiple endogenous controls and dedicated hemolysis-adjusted normalization were not performed. Therefore, some of the observed group differences may still be influenced by sample-processing variability, and external validation in larger, standardized cohorts is required.

## 5. Conclusions

In this case–control cohort, miR-99a-5p showed consistent and clinically meaningful suppression in laryngeal cancer and provided strong diagnostic discrimination, remaining independently associated with cancer status in a multimarker model. miR-1246 did not significantly separate cases from controls but displayed a significant stage-related suppression, supporting a complementary role in within-cancer stratification. Larger, multi-center, standardized, and longitudinal studies are warranted to validate thresholds, assess calibrations, and determine whether these circulating miRNAs improve clinical decision-making beyond established clinicopathologic factors.

## Figures and Tables

**Table 1:** RT-qPCR target reporting details.

Target	Mature miRBase ID	Mature miRBase Accession	Mature Sequence (5′→3′)	Assay Format
miR-99a-5p	hsa-miR-99a-5p	MIMAT0000097	AACCCGUAGAUCCGAUCUUGUG	TaqMan Advanced miRNA Assay
miR-1246	hsa-miR-1246	MIMAT0005898	AAUGGAUUUUUGGAGCAGG	TaqMan Advanced miRNA Assay
miR-16-5p	hsa-miR-16-5p	MIMAT0000069	UAGCAGCACGUAAAUAUUGGCG	TaqMan Advanced miRNA Assay

**Table 2:** Baseline clinical and demographic characteristics by stage group (early I–II vs. advanced III–IV).

Variable	Early (I–II)	Advanced (III–IV)	*p*-Value	Test
Age, years	66.9 ± 7.5	63.7 ± 8.7	0.203	*t*-test
Male sex	14/16 (87.5%)	42/45 (93.3%)	0.599	Fisher’s exact
Urban residence	9/16 (56.2%)	17/45 (37.8%)	0.323	χ^2^
T4 primary tumor	0/16 (0.0%)	20/45 (44.4%)	0.003	χ^2^
N+ nodal disease	0/16 (0.0%)	13/45 (28.9%)	0.014	Fisher’s exact
Distant metastasis (M1)	0/16 (0.0%)	2/45 (4.4%)	1	Fisher’s exact
Radiotherapy received	10/12 (83.3%)	30/32 (93.8%)	0.297	Fisher’s exact
Chemotherapy received	2/7 (28.6%)	15/26 (57.7%)	0.225	Fisher’s exact
Comorbidity count	4 [1–4]	4 [3–5]	0.147	Mann–Whitney U

M1, distant metastasis; N+, nodal involvement; and T4, tumor stage 4 primary.

**Table 3:** Baseline demographic and clinical characteristics of controls and LSCC cases.

Variable	Controls (*n* = 14)	LSCC Cases (*n* = 61)	*p*-Value	Test
Age, years	60.9 ± 7.7	64.5 ± 8.4	0.136	Welch’s *t*-test
Male sex	10/14 (71.4%)	56/61 (91.8%)	0.057	Fisher’s exact
Urban residence	8/14 (57.1%)	26/61 (42.6%)	0.381	Fisher’s exact
Comorbidity count	2 [1–3]	4 [2–5]	0.008	Mann–Whitney U
Active infection/acute inflammatory disease at sampling	0/14 (0.0%)	0/61 (0.0%)	—	By design

**Table 4:** Case–control comparison of circulating miRNAs.

miRNA	Controls (*n*)	Cancer (*n*)	ΔCt Controls Median [IQR]	ΔCt Cancer Median [IQR]	*p* (ΔCt)	Fold-Change (2^−ΔΔCt^) Cancer vs. Control Median [IQR]	*p* (Fold-Change)
miR-99a-5p	14	53	3.184 [2.142–3.708]	5.308 [4.139–6.864]	<0.001	0.200 [0.068–0.449]	<0.001
miR-1246	14	49	4.492 [2.869–5.511]	5.463 [3.816–6.824]	0.09	0.465 [0.181–1.457]	0.09

Ct, cycle threshold; ΔCt = Ct(target) − Ct(miR-16); ΔΔCt = ΔCt(sample) − meanΔCt(controls); and IQR, interquartile range.

**Table 5:** Stage-stratified miRNA expression within cancer (early I–II vs. advanced III–IV).

miRNA	Early (*n*)	Advanced (*n*)	ΔCt Early Median [IQR]	ΔCt Advanced Median [IQR]	*p* (ΔCt)	Fold-Change Early Median [IQR]	Fold-Change Advanced Median [IQR]	*p* (Fold-Change)
miR-99a-5p	12	38	3.788 [2.608–6.025]	5.332 [4.245–7.034]	0.052	0.575 [0.123–1.301]	0.197 [0.061–0.417]	0.052
miR-1246	13	33	4.233 [3.109–5.372]	5.820 [4.502–6.972]	0.01	1.091 [0.495–2.378]	0.363 [0.163–0.906]	0.01

IQR, interquartile range; ΔCt = Ct(target) − Ct(miR-16); and fold-change computed by 2^−ΔΔCt^ relative to controls.

**Table 6:** Treatment subgroup analysis within cancer: chemotherapy exposure (fold-change).

miRNA	No Chemotherapy (*n*)	Fold-Change Median [IQR]	Chemotherapy (*n*)	Fold-Change Median [IQR]	*p*-Value (Mann–Whitney)
miR-99a-5p	14	0.438 [0.206–0.600]	12	0.135 [0.086–0.259]	0.017
miR-1246	16	1.161 [0.260–3.362]	9	0.363 [0.200–0.752]	0.183

IQR, interquartile range; fold-change computed by 2^−ΔΔCt^ relative to controls.

**Table 7:** Spearman correlations between miRNA ΔCt values and clinical variables (within cancer).

Variable 1	Variable 2	Spearman ρ	*p*-Value
miR-99a-5p ΔCt	miR-1246 ΔCt	0.494	0.001
miR-99a-5p ΔCt	Age	−0.043	0.783
miR-99a-5p ΔCt	Stage (I–IV numeric)	0.191	0.221
miR-99a-5p ΔCt	Comorbidity count	−0.172	0.271
miR-1246 ΔCt	Age	−0.254	0.101
miR-1246 ΔCt	Stage (I–IV numeric)	0.278	0.071
miR-1246 ΔCt	Comorbidity count	0.045	0.773
Age	Stage (I–IV numeric)	−0.427	0.004
Age	Comorbidity count	0.414	0.006
Stage (I–IV numeric)	Comorbidity count	−0.221	0.156

ΔCt = Ct(target) − Ct(miR-16); ρ, Spearman rank correlation coefficient.

**Table 8:** Diagnostic performance (ROC) for distinguishing cancer patients vs. controls using ΔCt.

miRNA (Predictor: ΔCt)	Controls (*n*)	Cancer (*n*)	AUC	95% CI (Bootstrap)	Optimal ΔCt Cut-Off (Youden)	Sensitivity	Specificity	*p* (Group Diff; Mann–Whitney)
miR-99a-5p	14	53	0.842	[0.744, 0.931]	4.018	77.40%	92.90%	<0.001
miR-1246	14	49	0.65	[0.498, 0.782]	5.344	59.20%	71.40%	0.09

AUC, area under the ROC curve; CI, confidence interval; and ΔCt = Ct(target) − Ct(miR-16).

**Table 9:** Multimarker miRNA panel for discriminating cancer patients vs. controls (logistic model + internal validation).

Predictor	β	SE	OR (95% CI)	*p*
ΔCt_miR99a	0.635	0.236	1.89 (1.19–3.00)	0.007
ΔCt_miR1246	0.111	0.195	1.12 (0.76–1.64)	0.57

N = 59 (45 cancer, 14 controls). Predictors: ΔCt(miR-99a); ΔCt(miR-1246) (ΔCt = Ct(miRNA) − Ct(miR-16)). Model: Multivariable logistic regression (MLE). Internal validation: Bootstrap optimism correction (B = 400 resamples) for AUC + mean calibration slope.

**Table 10:** Stage contrast for the suppression score.

Stage Group	*n*	Mean Score ± SD	Mean Difference (Adv − Early)	*p* (Welch’s *t*-Test)	Hedges g
Early (I–II)	12	−0.675 ± 0.921			
Advanced (III–IV)	31	0.148 ± 1.126	0.823 (95% CI 0.135–1.512)	0.021	0.752

SD, standard deviation.

**Table 11:** Robust regression on the suppression score (HC3 SE).

Predictor	Model A β (SE)	Model A *p*	Model B β (SE)	Model B *p*
stage_num	0.643 (0.294)	0.029	0.922 (1.206)	0.445
chemotherapy		−8.579 (10.377)	0.408	
stage_num × chemotherapy		2.250 (3.248)	0.489	
T4	−1.061 (0.528)	0.044	−1.838 (3.333)	0.581
node-positive disease	0.245 (0.391)	0.53	0.352 (0.839)	0.674
age	−0.021 (0.025)	0.411	−0.037 (0.062)	0.545
comorbidity_count	0.071 (0.119)	0.552	0.281 (0.192)	0.145

Model A: N = 43 (minimal missingness). Model B (exploratory chemo interaction): N = 19 (chemotherapy recorded), including stage × chemo term; β, regression coefficient; HC3 SE, heteroskedasticity-consistent (HC3) standard errors; *p*, *p*-value; SE, standard error; stage_num, numeric stage variable; and T4, primary tumor stage 4.

## Data Availability

The data presented in this study are available on request from the corresponding author. The data are not publicly available due to privacy and ethical restrictions.
